# Supplementary material for: Premorbid Comorbidities as Predictors of Outcome in Ischemic Posterior Fossa Stroke: A Retrospective Evaluation Using the Age-Adjusted Charlson Comorbidity Index
Source: Brain Sci. 2025 Aug 21;15(8):892. doi: 10.3390/brainsci15080892 (PMC12384148; doi:10.3390/brainsci15080892)
Supplement: Supplementary file 1 [file brainsci-15-00892-s001.zip › brainsci-3796334-supplementary.pdf]

Sup. Tab. 1: Frequencies and baseline descriptive statistics of the Patients group with aCCI 0-4

Variables (n=114)	Value in Total	mRS at discharge 0-1 (n=73)	mRS at discharge ≥2 (n=41)	P value
Age at Indexevent*	64,5 (55-71)	68 (54-72)	63 (56-69)	0,464
Sex (female)	42 (36,8%)	30 (41.1%)	12 (29,3%)	0,212
Duration of hospital stay*	9 (6-13)	8 (6-11)	14 (8-23)	<0,001
Risk Factors				
• Diabetes	19 (16,7%)	12 (16,4%)	7 (17,1%)	0,591
• Previous Stroke	13 (11,4%)	8 (11,0%)	5 (12,2%)	0,717
• Atrial fibrilation	3 (2,6%)	1 (1,4%)	2 (4,9%)	0,819
• Hypertension	75 (65,8%)	49 (67,1%)	26 (63,4%)	0,241
• Overweight/Obesity (BMI>25 mg/kg/cm2)	65 (57,0%)	45 (61,6%)	20 (48,8%)	0,867
• Smoking	17 (14,9%)	12 (16,4%)	5 (12,2%)	0,977
• Alkohol	7 (6,1%)	1 (1,4%)	6 (14,6%)	0,675
• Drugs	0 (0,0%)	0 (0.0%)	0 (0.0%)	-
• Hyperlipidemia	33 (28,9%)	22 (30,1%)	11 (26,8%)	0,271
NIHSS at presentation*	1 (0-4)	1 (0-2)	4 (2-9)	<0,001
GCS at presentation*	15 (15-15)	15 (15-15)	15 (15-15)	0,001
mRS at presentation*	2 (1-3)	1 (1-2)	3 (2-4)	<0,001

Sup. Tab. 2: Frequencies and baseline descriptive statistics of the Patients group with aCCI ≥5

Variables (n=172)	Value in Total	mRS at discharge 0-1 (n=38)	mRS at discharge ≥2 (n=34)	P value
Age at Indexevent*	76 (71-82)	75,5 (73-82)	76,5 (71-82)	0,573
Sex (female)	30 (41,7%)	16 (42,1%)	14 (41,2%)	0,588
Duration of hospital stay*	12 (7-20)	9 (6-13)	14 (9-25)	0,118
Risk Factors				
• Diabetes	30 (41,7%)	16 (42,1%)	14 (41,2%)	0,382
• Previous Stroke	22 (30,6%)	13 (34,2%)	9 (26,5%)	0,886
• Atrial fibrilation	19 (26,4%)	10 (26,3%)	9 (26,5%)	0,021
• Hypertension	56 (77,8%)	31 (81,6%)	25 (73,5%)	0,549
• Overweight/Obesity (BMI>25 mg/kg/cm2)	43 (59,7%)	23 (60,5%)	20 (58,8%)	0,273
• Smoking	7 (9,7%)	4 (10,5%)	3 (8,8%)	0,508
• Alkohol	6 (8,3%)	1 (2,6%)	5 (14,7%)	0,673
• Drugs	0 (0,0%)	0 (0,0%)	0 (0,0%)	-
• Hyperlipidemia	41 (56,9%)	21 (55,3%)	20 (58,8%)	0,774
NIHSS at presentation*	3 (1-4)	1 (1-3)	4,5 (3-9)	0,002
GCS at presentation*	15 (15-15)	15 (15-15)	15 (14-15)	0,206
mRS at presentation*	2,5 (1-3)	1 (1-2)	3 (3-4)	<0,001

Sup. Fig. 1: Distribution of aCCI-Score in posterior cranial fossa infarct cohorts

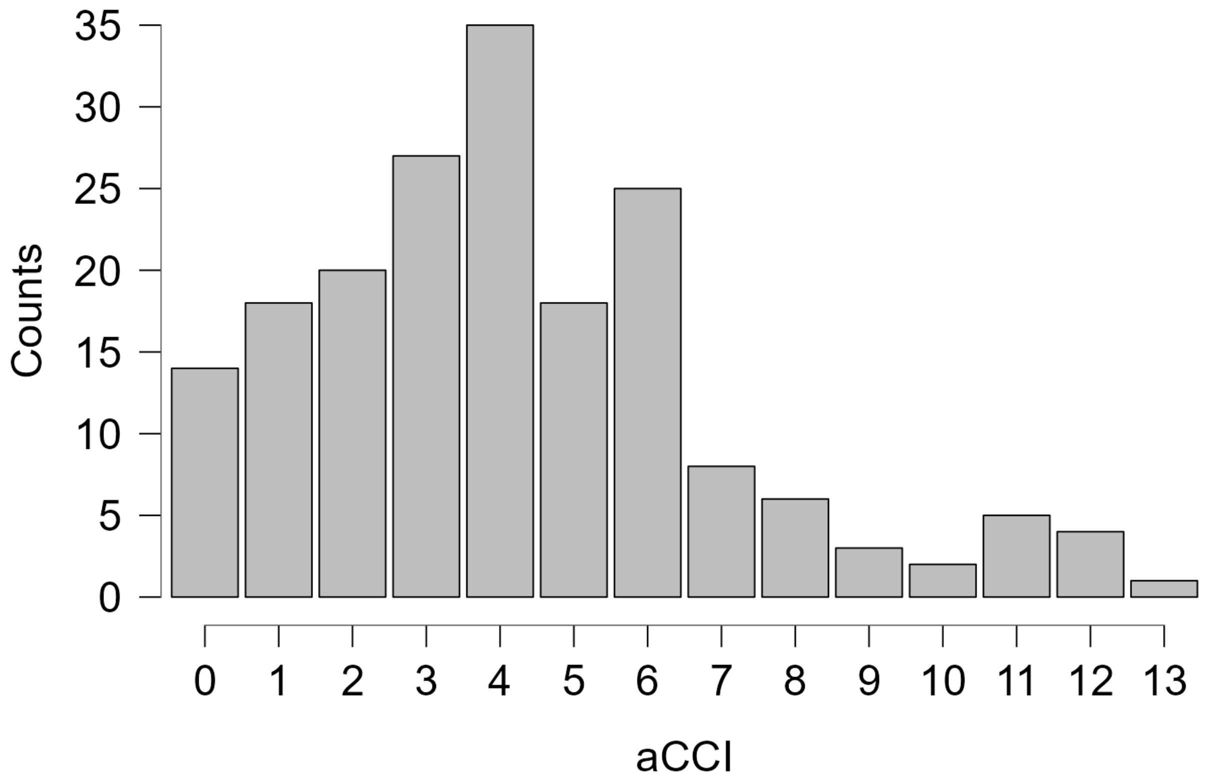

SEM Model:

Latent variables:

- CCI=~x1+x2
- NIH=~y1+y2+y3
- MRS=~y4+y5+y6

Regression:

- NIH~CCI
- MRS~CCI+NIH
- NIH~alpha*CCI
- MRS~direct*CCI+beta*NIH
- Indirect=alpha*beta
- Total :=indirect+direct
- Proportion :=indirect/total

X1=aCCI 0-4; X=aCCI ≥5;

Y1=NIHSS 0-6; Y2=NIHSS 7-41; Y3=NIHSS 42;

Y4=mRS 0-1; Y5=mRS 2-5; Y6=mRS 6.
